# Revision of total elbow arthroplasty due to humeral loosening with large bone defect using humeral allograft-prosthesis composite: A case report

**DOI:** 10.1016/j.ijscr.2025.111993

**Published:** 2025-10-03

**Authors:** Eleni Pappa, Tim Cresswell

**Affiliations:** a KAT General Hospital, Athens, Greece; b Royal Derby Hospital, Derby, UK

**Keywords:** Total elbow arthroplasty, Loosening, Bone defect, Allograft, Allograft-prosthesis composite, Revision, Rheumatoid arthritis, Case report

## Abstract

**Introduction and importance:**

Revision of total elbow arthroplasty is a challenging procedure, especially when associated with humeral bone deficiency. The purpose of this case report is to highlight the successful management of humeral-sided loosening due to bone defects, using an allograft-prosthesis composite with a humeral bone allograft.

**Presentation of case:**

A 70-year-old female patient under medication for rheumatoid arthritis underwent revision of left total elbow arthroplasty due to major bone defect and loosening on the humeral side. The allograft-prosthesis composite method was used to address the bone defect and loosening of the humeral side by using humeral allograft as well as plating the host humeral bone.

**Clinical discussion:**

There were no post-operative complications. The radiographic assessment at her latest follow-up was unremarkable, along with a significant improvement on the functional scores and range of motion.

**Conclusion:**

The use of a humeral allograft is a valuable option for the management of loosened total elbow arthroplasty with significant bone loss. However, more studies need to be conducted to determine the long-term outcomes of revision surgery of total elbow arthroplasty with humeral bone loss.

## Highlights

• Revision of total elbow arthroplasty in RA patients with humeral-sided loosening and severe bone defect remains a major surgical challenge.
• Humeral loosening in TEAs is not as common as the ulnar-sided loosening.
• A humeral allograft–prosthesis composite (APC) was successfully used to reconstruct the distal humerus.
• Compression plate fixation at the host–allograft junction provided stable integration and prevented non-union.
• The technique achieved pain-free range of motion and excellent short-term functional outcome without complications.

## 1 Introduction

Total elbow arthroplasty (TEA) is widely performed for the management of elbow joint damage caused mainly by post-traumatic arthritis and acute trauma, whereas the rheumatoid arthritis which was the main causing factor tends to be better managed with the current disease modifying factors [1]. On the one hand, the revision rate for TEA has been mentioned to be as high as 12 % for rheumatoid arthritis, while on the other hand it is almost 19 % for post-traumatic cases [4]. Semi-constrained prostheses are mainly used nowadays, as the fully constrained ones led to a high incidence of loosening, while the non-constrained prostheses signed frequent dislocations [2]. It is also mentioned in a recent study by Teytelbaum et al., that a humeral prosthesis with a relatively short flange relative to the stem length can also play a leading role in the increased revision rate of TEA, especially when rheumatoid arthritis is present [14].

One of the most major complications of either primary or revision TEA is the aseptic loosening of the components, where in that case bone defects are likely to be present either on the ulnar or more infrequently on the humeral side. The management of those bone defects is of outstanding importance, as many reconstruction options have been reported in the current literature including autologous impaction grafting, allografts, custom made prostheses or implants made for tumor cases such as megaprostheses [12]. We report the case of a female patient who underwent revision TEA using humeral allograft and a locking plate for the management of the humeral-sided loosening which was cause by a significant bone defect. Informed consent was obtained from the patient. This case report has been reported in line with the SCARE checklist [17].

## 2 Presentation of case

**Fig. 1:**
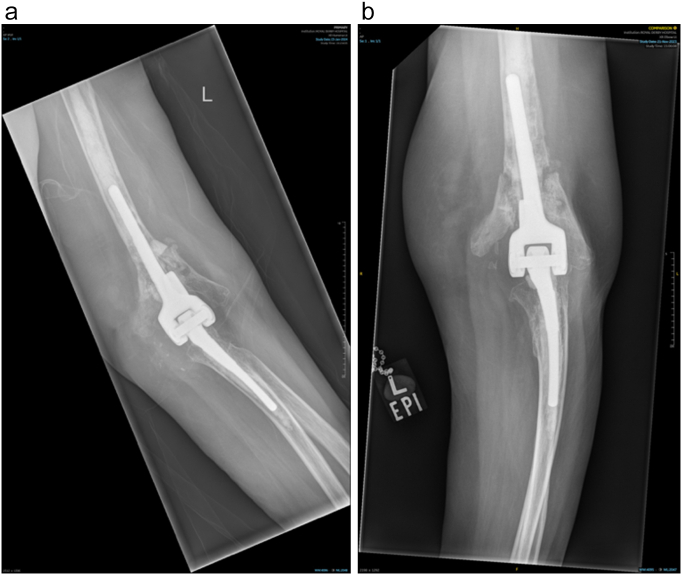
Anteroposterior [A] and lateral [B] radiographic views showing the initially revised TEA of the left elbow on the last radiographic assessment due to loosening and periprosthetic fracture, in 2023.

**Fig. 2:**
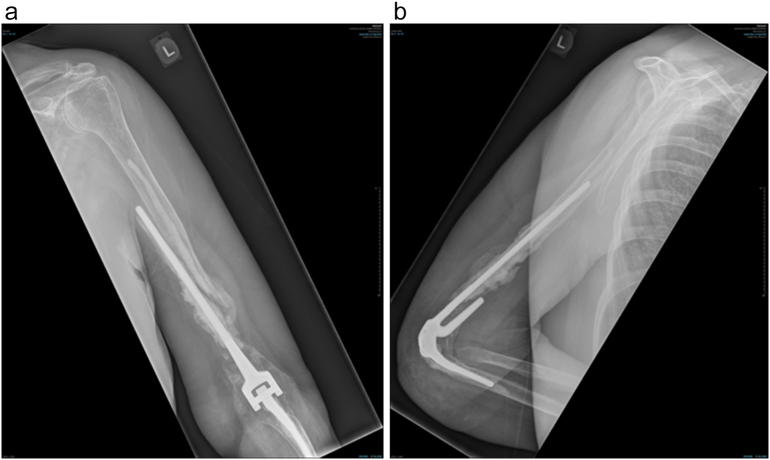
Pre-operative X-ray in 2024, after the previously revised TEA. Anteroposterior [A] and lateral [B] views show the re-loosening of the humeral prosthesis together with significant bone loss and complete perforation of the cortical bone. No ulnar loosening is present.

The patient is a 70-year-old female. She had undergone TEA of both elbows due to joint destruction because of rheumatoid arthritis, and post-traumatic arthritis following an intra-articular fracture in 2006. She had a Coonrad-Morrey TEA in 2007. Since then, revision surgery had been performed in 2017 to the right elbow because of ulnar-sided loosening, but on the left elbow she had undergone revision on 2023 due to humeral-sided loosening and a peri-prosthetic fracture [Fig. 1]. In follow-up of the left sided TEA, loosening of the humeral component seemed to progress and her daily living activities were now impaired due to increased pain and impaired range of motion during manual activities. The patient was investigated for infection, with negative serum markers (WBC, CRP, ESR). Preoperative radiographic examinations showed re-loosening of the humeral stem and complete perforation of the cortical bone proximally to the stem. However, there was no loosening of the ulnar side [Fig. 2].

In order to address the progressive humeral-sided bone defect, it was decided to perform a revision of the TEA of the left elbow with humeral bone allograft material provided by Joint Operations. For this specific case, the distal humeral bone defect was managed using allogeneic humeral diaphysis together with a locking plate on the host humeral bone on the proximal side.

## 3 Surgical technique

**Fig. 3:**
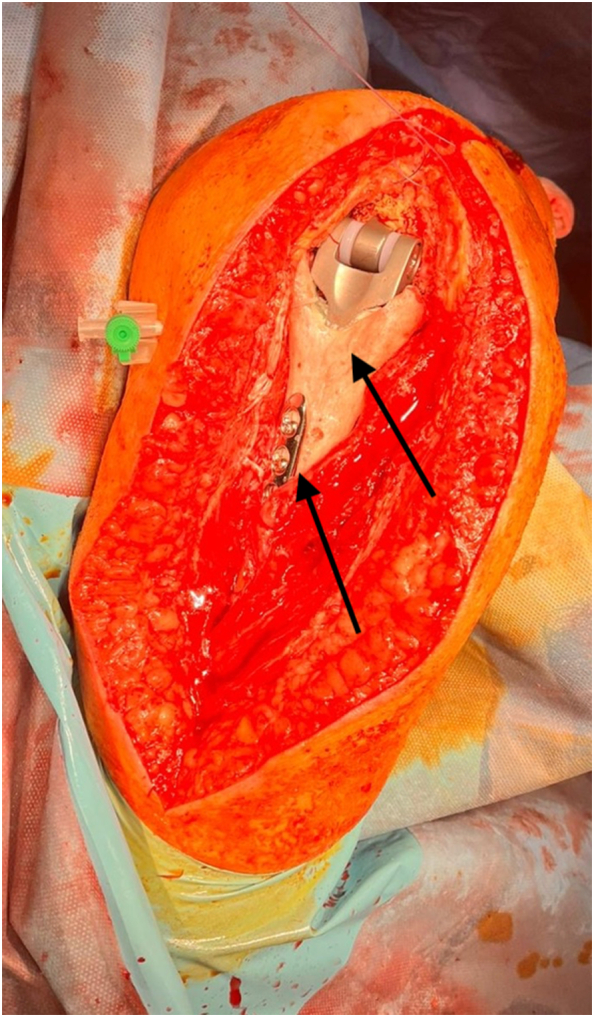
Intraoperative procedure. Under general anesthesia and lateral decubitus position with posterior approach soft tissue is dissected to expose the ulnar and radial nerve and the final APC is positioned with a compression plate and cement to the host humerus. Arrows are showing the humeral allograft and the plate.

**Fig. 4:**
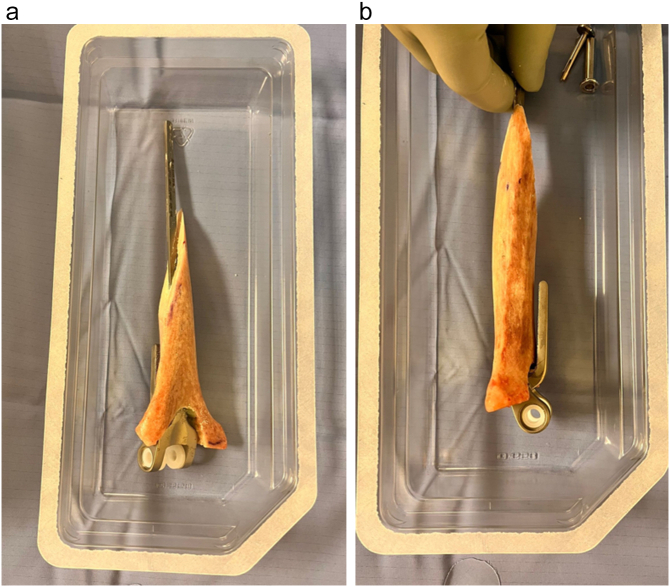
Humerus diaphysis allograft prosthesis composite with cemented final humeral prosthesis in place after gradual processing with the stem trial and cuts according to the size of the bone defect.

**Fig. 5:**
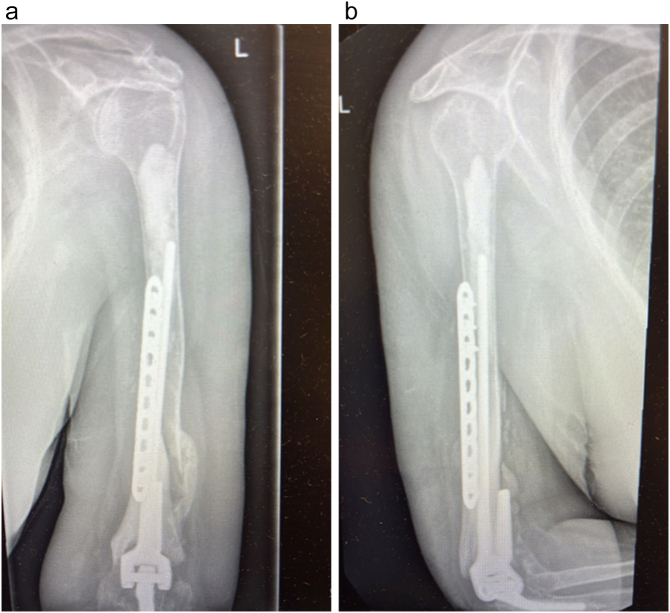
Post-operative X-ray and one month follow up. In anteroposterior [A] and lateral [B] views the area around the humeral stem is stable with no loosening present or any early resorption of the allograft.

**Fig. 6:**
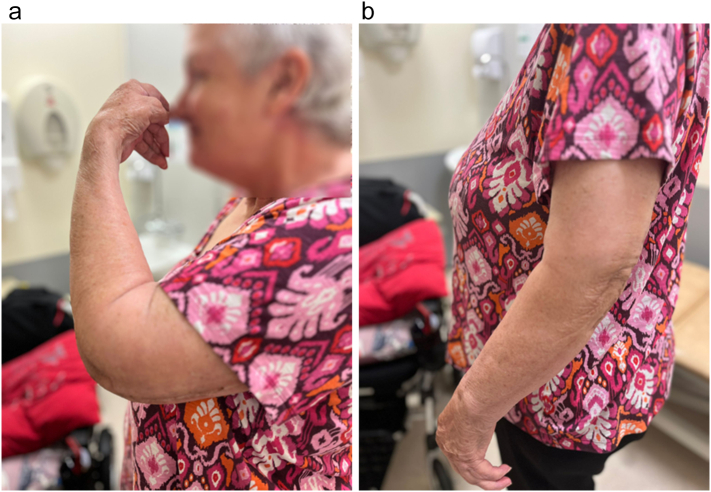
Clinical presentation on one month follow up. Range of motion of the left elbow achieving 120° in flexion and 30° of extension.

**Fig. 7:**
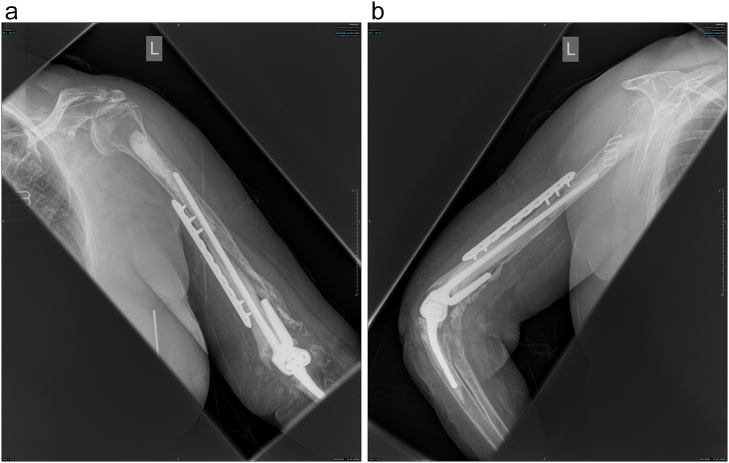
Postoperative radiographs, at 6 months, demonstrating the intact humeral allograft-plate-construct, no evidence of loosening of the humeral stem, and osseous union at the step-cut allograft humeral-host interface.

Surgery was performed under general anesthesia in lateral decubitus position with posterior triceps splitting approach. The triceps was functional without any defects however the TEA humeral prosthesis was found exactly under the subcutaneous tissue as a significant bone defect of the distal humerus was present. Both the ulnar and radial nerve where identified and found intact. Despite the loosening and cortical perforation, no findings suggestive of infection were present, however the results of culture tests of the soft tissue of the surgical field were found negative. The humeral prosthesis was resected from the proximal humerus together with the existing cement mantle en bloc, however bone defect was marked extending to the distal humerus, approximately 8 cm medially and 11 cm laterally in length. For the reconstruction of the distal humerus, we used allografted distal humerus provided by Joint Operations. The length and shape of the allograft were processed according to the bone defect size together with the stem trial rasps. Then, the final humeral implant was cemented within the allograft on the surgical table and the final construct was cemented within the host humeral diaphysis with the addition of a compression plate which was positioned on the junction of the host bone and the humeral allograft, forming in that way an allograft prosthesis composite [APC]. The 3.5 mm plate fixed to the APC was slid on the posterior of the proximal humerus, and as per AO principles, it was fixed with 3 non-locking screws proximally and 2 non-locking screws distally, through the incision of the posterior approach, providing absolute stability to the junction of the construct, so as to prevent any non-union [Fig. 3, Fig. 4]. Gentamycin impregnated bone cement (Palacos R + G, Hereus) when creating APC, together with intraarticular administration of 1 g of Gentamycin, were used to inhibit any infection. Because either no loosening of the ulnar side or any wear of the polyethylene were present, there was no revision of the ulnar side or polyethylene exchange. After the connection of the humeral with the ulnar prosthesis, we confirmed that a full range of elbow motion was present. Post-operative x-rays showed good placement of the APC and the plate [Fig. 5]. During the 8 weeks follow up, the post-operative course was uneventful and the Oxford Elbow Score was found 42. Range of motion was 120 of flexion and 30 of extension (30–120 degrees), while the patient was completely pain free without any complications including infection, having already returned to her previous daily activities [Fig. 6]. On her latest follow-up, 6 months post-operatively, the patient still has a pain-free range of motion of 120 degrees of flexion and 30 degrees of extension, maintaining her Oxford Elbow score. Also, there are no signs of infection, whereas the APC healing is monitored through radiographs showing uneventful graft incorporation and a stable plate-and-screw fixation, with no re-loosening or non-union present [Fig. 7].

## 4 Clinical discussion

In this case report, good results were achieved regarding the management of loosening of the humeral prosthesis of the TEA by the reconstruction of the significant bone defect with APC and plate fixation with the humerus of the host, using a humerus allograft. The allogeneic humeral bone was firmly fixed with the humerus with a locking plate. Laumenerie et al. in their case series in 2023 highlighted the successful use of APCs in the treatment of TEA loosening with bone loss, as their study resulted in satisfactory medium-term results and stable fixation of the prostheses on follow-up [7].

Many surgical options exist regarding the management of aseptic loosening with bone defect in TEA. Autologous impaction grafting, allograft bone, custom made TEAs as well as mega-prostheses have all been used for the treatment of the bone defects.

One the one hand, Johnson et al. treated the aseptic loosening of TEA with impaction grafting together with revision of the humeral side, however on that case there was no cortical perforation of the humerus and only medullary defect was present [5]. Rhee et al. in their case series also used the impaction grafting technique leading to the significant improvement of the post-operative Mayo Elbow Performance Scores (MEPS) of their patients [13].

On the other hand, Loebenberg et al. in their case series also reported their experience with impaction bone grafting in order to address the osteolysis of both the humeral and ulnar side, mentioning improvement in the post-operative range of motion and functional performance [9].

Furthermore, autogenous and allogeneic bone grafting have been used according to the current literature. Kajiyama et al. in their case report managed the bone defect of the humeral side in their revision case by using an APC with plate construct but the allogeneic bone was a femur and not a humerus as in our case. Unfortunately, diaphyseal allograft bones are not always available for use. For instance, Kajiyama et al. in their case report did not have available humeral allografts from the industry [6]. Furthermore, Lee et al. in their retrospective study in 2021, addressed the large bone defects on revision surgery of TEA with autogenous fibular strut and iliac bone grafts achieving a good union rate [8].

Thus, the advantage of the use of humeral bone instead of femoral bone as an allograft could be highlighted as the less bulky APC which is constructed, in contrast with the use of femoral or fibular allograft. Mansat et al. also reported in their case series good results by using the step-cut technique, circular wiring and plates for the fixation of APCs, leading to the confirmation of the usefulness of these constructs [10]. However, in our case wiring was not used as the stability of the APC was enhanced by only the locking plate. Furthermore, Morrey et al. highlighted the importance of the allograft bone to being originated by the same site of the defect, such as the distal humerus in our case, leading to better functional results. Wiring has also been used instead of plating of the APC with the host bone, as indicated by Morrey et al., guaranteeing also acceptable functional results. In our case, the plate was considered as the best means of stability of the APC with the host bone [11]. Cheema et al. in their case series in 2023 used the same technique of APC, including humeral allograft bone and compression plating with or without cables and wiring, in order to increase the stability between the host bone and the construct and diminish the rate of post-operative non-union, achieving the salvage of approximately two-thirds of the elbows included in their study from the severe and debilitating condition of humeral loosening of TEA [3]. Last but not least, even personalized 3D-printed prostheses have been mentioned in the literature accompanied or not by the Masquelet technique, regarding the management of severe bone loss in post-traumatic total elbow arthroplasty [15,16].

Regarding the limitations of this study, it has to be highlighted that it is a case report with short-term outcomes regarding the follow up of the patient of only 6 months. However, despite the limited follow-up, no infection or re-loosening because of allograft absorption is present.

## 5 Conclusion

In this case, humeral component loosening of total elbow arthroplasty in a patient with rheumatoid arthritis was successfully reconstructed using a humeral allograft–prosthesis composite combined with a compression plate fixation. This approach provided excellent short-term functional outcomes. However, long-term follow-up and additional studies are needed to confirm the reliability and effectiveness of this advanced technique in managing humeral bone loss during revision TEA.

## Patient consent

Verbal and written consent were received by the patient prior to the preparation of the manuscript.

## Ethical approval

It was not required from our IRB.

## Guarantor

Mr. Tim Cresswell.

## Funding

No funding was disclosed by the authors.

## Author contribution

Mr. Tim Cresswell: provided medical treatment and follow-up, provided the data of the manuscript, and made grammar and spelling language editing and also supervised the manuscript preparation. Ms. Eleni Pappa: wrote the manuscript, did scientific research on the topic, organised the data provided.

## Declaration of competing interest

The authors have no conflicts of interest to disclose.
